# Tregs protect against combination checkpoint blockade toxicity induced by Tph and B cell interactions

**DOI:** 10.1172/JCI174724

**Published:** 2024-05-02

**Authors:** Alyssa Duffy, Maryam I. Azeem, Smriti Kanangat, Melinda Yushak, David Lawson, Madhav V. Dhodapkar, Kavita M. Dhodapkar

**Affiliations:** 1 Department of Hematology and Medical Oncology and; 2 Department of Pediatrics, Division of Hematology and Oncology, Emory University, Atlanta, Georgia, USA.

**Keywords:** Immunology, Oncology, Cancer immunotherapy, Immunotherapy

**To the editor:** Immune-related adverse events (irAEs) have emerged as a challenge for combination checkpoint blockade (CCB) in cancer (1). CTLA-4 and PD-1 checkpoints affect both B and T cell tolerance, and both cells have been implicated in irAEs. Early changes in B cells (2) as well as baseline features of CD4^+^ T cells (3) have been correlated with irAEs following CCB therapy. However, the nature of specific T cells that help pathogenic B cells and the underlying mechanisms remain unknown.

We analyzed blood specimens before and after the first cycle of CCB therapy in a cohort of patients with melanoma undergoing CCB (clinical characteristics in Supplemental Table 1; supplemental material available online with this article; https://doi.org/10.1172/JCI174724DS1). CCB therapy led to an increase in CD21^lo^CD11c^+^ B cells as well as plasmablasts and a decline in circulating B cells in patients who developed high-grade irAEs (HG-irAEs) (Supplemental Figure 1, A–C) (2). The phenotype of expanded CD21^lo^CD11c^+^ B cells was consistent with the presence of CXCR5-T-bet^+^ B cells implicated in extrafollicular B cell responses and autoimmunity (Supplemental Figure 1D). FlowSOM analysis identified 2 distinct metaclusters (MCs) of Ki-67^+^CD4^+^ T cells (MC3 and MC8) and a Ki-67^+^CD8^+^ MC (MC4) that underwent early proliferation following therapy (Supplemental Figure 1, E and F). Notably, MC6, which contained CXCR5^+^PD1^+^CD4^+^ T cells (consistent with T follicular helper cells), did not change following therapy (Supplemental Figure 1, F and G). Proliferating CD4^+^ T cells expressed ICOS but not CXCR5 (Supplemental Figure 1H), similar to T peripheral helper (Tph) cells (4). Next, we tested the capacity of purified ICOS^+^ or ICOS^–^CD4^+^ T cells expanded in CCB-treated patients to induce B cell differentiation in T/B cocultures. Addition of ICOS^+^CD4^+^ T cells (but not ICOS^–^CD4^+^ T cells) to B cells led to the induction of CD27^+^CD38^+^ plasmablasts (Figure 1A) and human IgG secretion (Figure 1B). Plasmablast induction was significantly greater in cocultures with memory versus naive B cells (Figure 1C). T cell subsets were adoptively transferred with human B cells into MISTRG6 mice and monitored for the development of plasmablasts and Ig secretion. Coinjection of B cells with ICOS^+^CD4^+^ T cells (but not ICOS^–^ counterparts) led to plasmablast differentiation (Figure 1, D and E) and human IgG production (Figure 1F). Together, these data demonstrate that CCB therapy leads to proliferation of CD21^lo^CD11c^+^ B cells as well as ICOS^+^ Tph-like cells that help B cells, illustrating enhanced T/B cooperation following therapy.

ICOS^+^CD4^+^ T cell activation was associated with secretion of sCD40L, IL-21, and IFN-γ (Supplemental Figure 2, A–C). Plasmablast induction in T/B cocultures was inhibited by the blockade of CD40 (Supplemental Figure 2D), IL-21 (Supplemental Figure 2E) and IFN-γ–mediated signaling (Supplemental Figure 2F). The increase in circulating ICOS^+^CD4^+^ T cells was similar in patients with and without HG-irAEs (Figure 1G). However ICOS^+^ T cells from patients with HG-irAEs had greater capacity to provide help to B cells, suggesting functional differences in ICOS^+^ T cells (Figure 1H). FlowSOM analysis of ICOS^+^CD4^+^ T cells (Supplemental Figure 3, A and B) revealed that patients without HG-irAEs had a higher proportion of MC6 and MC10 in the posttreatment ICOS^+^CD4^+^ T cells, which coexpressed FOXP3 and CD25, consistent with a Treg phenotype (Figure 1I). Patients with HG-irAEs showed a trend of a higher proportion of a CXCR3+ MCs (MC7) and expressed PD1 at baseline (Supplemental Figure 3, B and C). Depletion of Tregs from ICOS^+^CD4^+^ T cells led to increased plasmablasts (Figure 1J). Together, these data suggest that Tregs may suppress T/B interactions in patients without HG-irAEs.

The proportion of proliferating CD4^+^ and CD8^+^ T cells did not differ between patients with or without HG-irAEs (2) (Supplemental Figure 3D). However, patients with HG-irAEs had higher expression of CXCR3 in proliferating CD4^+^ and CD8^+^ T cells and CD21^lo^CD11c^+^ B cells but not myeloid cells (Supplemental Figure 3E). The expression of CXCR3 in ICOS^+^ CD4^+^ T cells correlated with a more effector/activated phenotype, with higher expression of T-bet and HLA-DR (Supplemental Figure 3F).

In summary, we show that CCB therapy leads to expansion of ICOS^+^ Tph cells and CD21^lo^CD11c^+^ B cells. Both cell types express PD-1 and play an outsized role in human autoimmunity (4). However, irAE development depends on the induction of Tregs, which inhibit Tph/B interactions. Expansion of ICOS^+^ T cells by CTLA-4 blockade has also been implicated in mediating antitumor effects, suggesting potential overlap with mechanisms underlying irAEs. CXCR3 has been previously implicated in autoimmune tissue homing (4). Strengths of this work are analysis of uniformly treated patients and inclusion of in vitro and in vivo studies to dissect mechanisms. Limitations include small sample size and lack of analysis of autoimmune tissues. Underlying mechanisms may also differ by specific types of irAEs, which were not studied here. Blockade of specific pathways, such as IL-21/IL-21R–mediated signaling and boosting Tregs, may provide novel strategies to prevent or treat CCB-induced irAEs.

Melanoma biospecimens from patients undergoing CCB therapy were collected after patients provided written informed consent. Studies were approved by the Emory University institutional review board. Details regarding the methods are provided in the Supplemental Methods.

## Supplementary Material

Supplemental data

Supporting data values

## Figures and Tables

**Figure 1:**
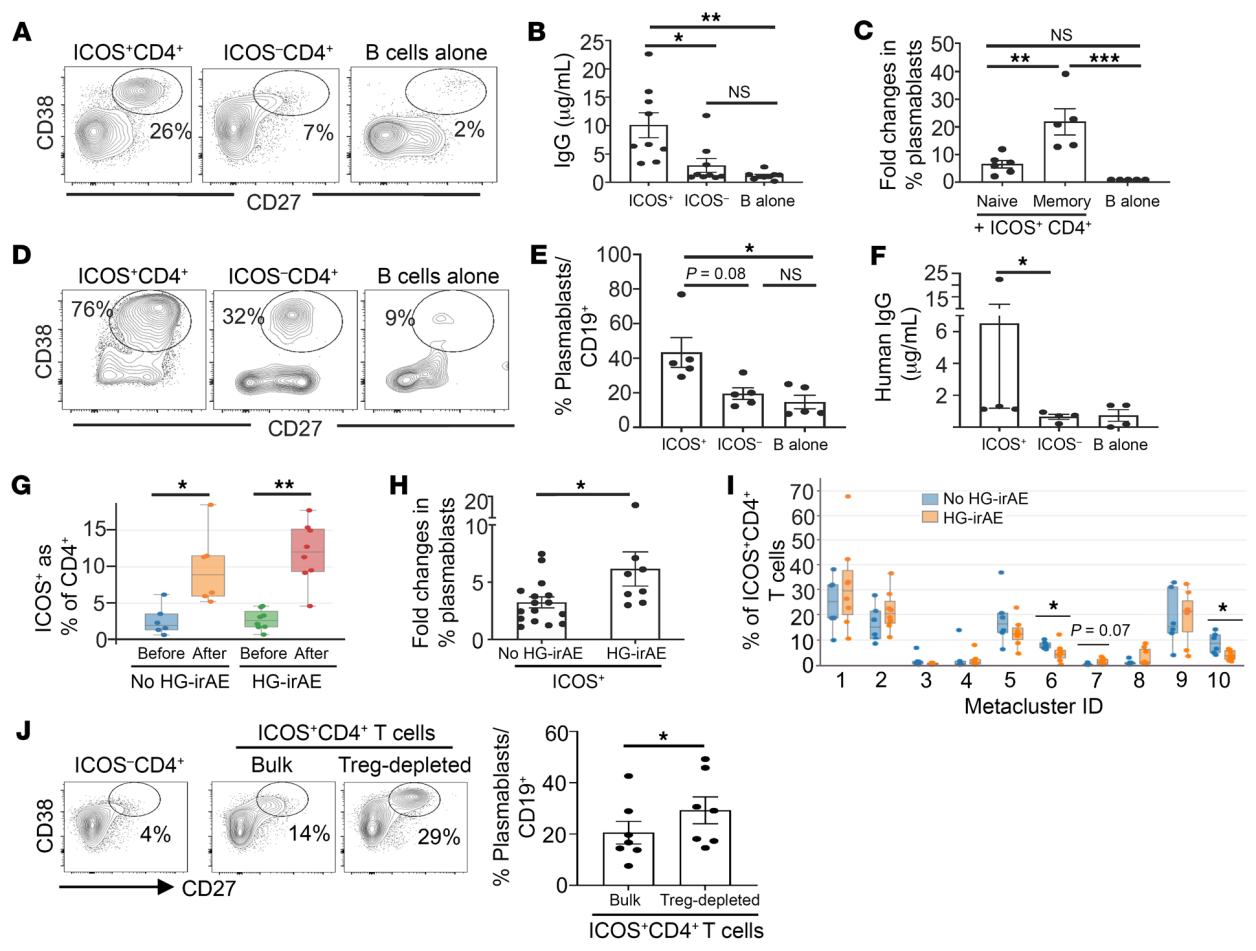
Mechanisms of T/B interactions. (**A**–**C**) T/B cocultures. Purified B cells were cultured alone or with flow-sorted ICOS^+^CD4^+^ and ICOS^–^CD4^+^ T cells from patients with melanoma after CCB treatment (Supplemental Methods). (**A**) Representative flow plot of CD38^+^CD27^+^ plasmablast. (**B**) Human IgG levels by ELISA (*n* = 9). Kruskal-Wallis with Dunn’s multiple comparisons correction. (**C**) Effect of B cell subsets. Flow-sorted posttreatment T cells were cultured with memory(*n* = 5) or naive B cells (*n* = 6). Bar graphs indicate fold change compared with cultures with B cells alone. One-way ANOVA with Tukey’s multiple test correction. (**D**–**F**) Human T/B interactions in vivo. Sorted ICOS^+^CD4^+^ or ICOS^–^CD4^+^ T cells from patients with melanoma (*n* = 5) were injected into MISTRG6 mice along with purified human B cells (Supplemental Methods). (**D**) Representative flow plots of plasmablast frequency in the spleen at 2 weeks after injection, as quantified in **E**. One-way ANOVA with Dunn’s multiple correction. (**F**) Human IgG levels detected by ELISA in mice from **E**. Wilcoxon’s matched-pairs signed-rank test. (**G**) Bar graph shows expansion of ICOS^+^CD4^+^ T cells after CCB therapy in patients with no HG-irAEs and those with HG-irAEs. Wilcoxon’s matched-pairs rank test. (**H**) B cells were cultured with flow-sorted ICOS^+^CD4^+^ and ICOS^–^CD4^+^ T cells from patients with HG-irAEs or no HG-irAEs. Bar graph shows fold change in plasmablasts in ICOS^+^ versus ICOS^–^ cocultures. Mann-Whitney. (**I**) FlowSOM performed on ICOS^+^CD4^+^ T cells obtained following 1 cycle of combination therapy identified 10 metaclusters (MCs). Bar graph shows proportions of these MCs in patients with HG-irAEs or no HG-irAEs. Mann-Whitney. (**J**) Depletion of Tregs in patients with no HG-irAEs leads to enhanced B cell activation by ICOS^+^CD4^+^ T cells. Purified B cells were cultured with ICOS^–^, bulk ICOS^+^, or Treg-depleted ICOS^+^CD4^+^ T cells from patients with no HG-irAEs. Dot plot shows plasmablast frequency as percentage of B cells. Bar graph shows data for 7 different experiments. Wilcoxon’s matched-pairs rank test. *P < 0.05, **P < 0.01, ***P < 0.001.

## Version 1 05/02/2024

In-Press Preview

## Version 2 06/17/2024

Electronic publication
