# Supplementary material for: Tregs protect against combination checkpoint blockade toxicity induced by Tph and B cell interactions
Source: J Clin Invest. 2024 May 2;134(12):e174724. doi: 10.1172/JCI174724 (PMC11178527; doi:10.1172/JCI174724)
Supplement: Supplemental data [file jci-134-174724-s073.pdf]

Supplementary Appendix

Duffy et al.

Table of Contents:

Supplementary Table 1

Supplementary Figures 1-3

Supplementary online methods

Supplementary Table 1 Patient Characteristics

	**No- HG irAEs; n=6**	**HG* irAEs; n=8**
**Median Age (yrs) (range)**	67 (54-76)	65 (44-75)
**Gender Male (%)**	5/6 (83)	4/8(50)
**Grade 3 irAE organ involved**	NA	GI (6), Skin, Kidney and Joints (1 each)

* HG= $\geq$ Grade 3 by common toxicity criteria, v5.

Supplementary Fig1

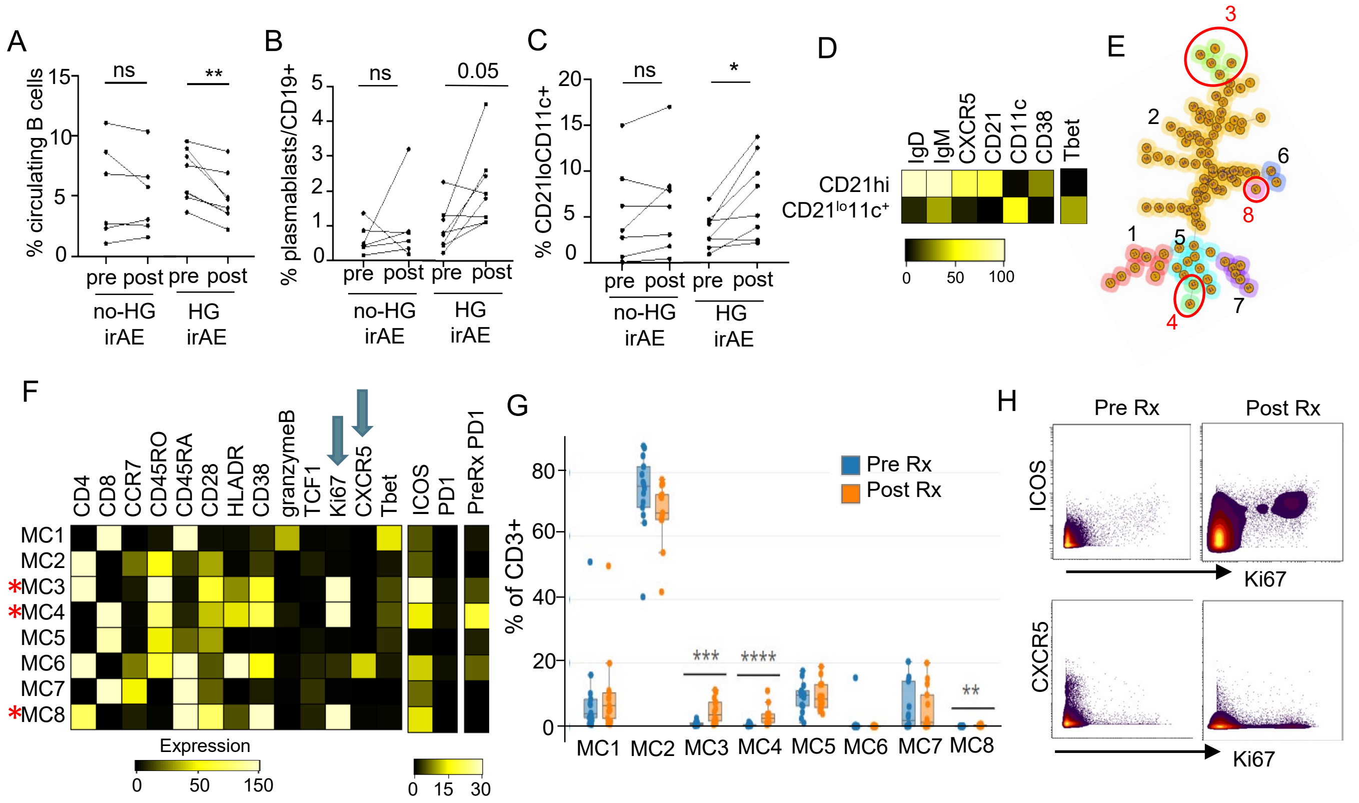

Suppl Fig1. Changes in T and B cells following combination checkpoint blockade and the development of irAEs. **(A-H)** Peripheral blood mononuclear cells (PBMCs) obtained from patients before and after one cycle of combination anti-PD1 and anti-CTLA-4 therapy (n=14) were analyzed by mass cytometry. **(A-C)** Graphs showing the frequency of circulating B cells **(A)**, plasmablasts **(B)**, and CD21^{lo}CD11c+ B cells **(C)** as a percentage of total B cells. Patients were grouped into cohorts with high-grade irAEs (CTC grades 3-4; n=8) or no high-grade irAEs (CTC grades 0-2; n=6). **(D)** Phenotype of CD21^{lo} B cells: Heatmap displays expression of IgD, IgM, CXCR5, CD21, CD11c, CD38, and T-bet on CD21^{hi} versus CD21^{lo}CD11c^{+} B cells. **(E-G)** Phenotype of T cells before/after therapy: **(E)** FlowSOM analysis of CD3+ T cells identified 8 distinct T cell metaclusters (MC)**.** Proliferating MCs that differ between pre and post-treatment specimens are circled. **(F)** Heatmap showing phenotype of the MCs (*identifies Ki-67+ proliferating subsets). **(G)** Bar graph showing proportion of each MC (as percentage of T cells) in patients before/after therapy. **(H)** Proliferating CD4+ T cells are ICOS+CXCR5-. Representative plots showing expression of ICOS and CXCR5 on proliferating CD4+ cells. *p<0.05, **p<0.01, ***p<0.001, ****p<0.0001, by 2-tailed Wilcoxon signed rank test.

Supplementary Fig2

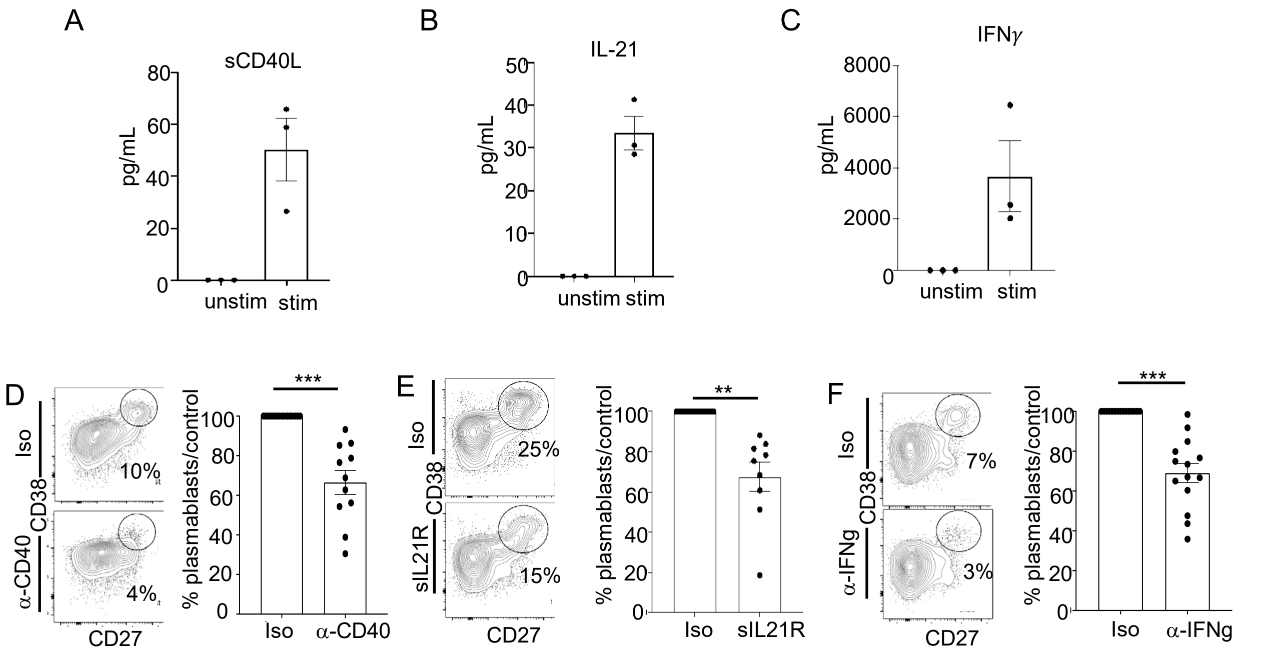

Suppl Fig2. Mechanisms of T cell help. **(A-C)** ICOS+CD4+ T cells were flow sorted from peripheral blood of melanoma patients after CCB treatment and stimulated with PMA/Ionomycin for 24 hrs. Cell supernatant was analyzed for presence of sCD40L **(A)**, IL-21 **(B)**, and IFNγ **(C). (D-F)** Flow-sorted ICOS+CD4+ T cells from post CCB treated melanoma patients were cultured with purified human B cells in the presence of isotype control or CD40 blocking antibodies **(D)**, soluble IL-21 receptor **(E)**, or interferon-gamma blocking antibodies **(F)** as described in the methods. One week later, proportion of plasmablasts were assessed in these cocultures. Dot plots in D-F show data from a representative patient and bar graphs show data from all patient samples as a proportion of isotype control. Each dot represents a unique patient sample. For all panels: *$p<0.05$, **$p<0.01$, ***$p<0.001$, ****$p<0.0001$, by 2-tailed Wilcoxon signed rank test.

Supplementary Fig3

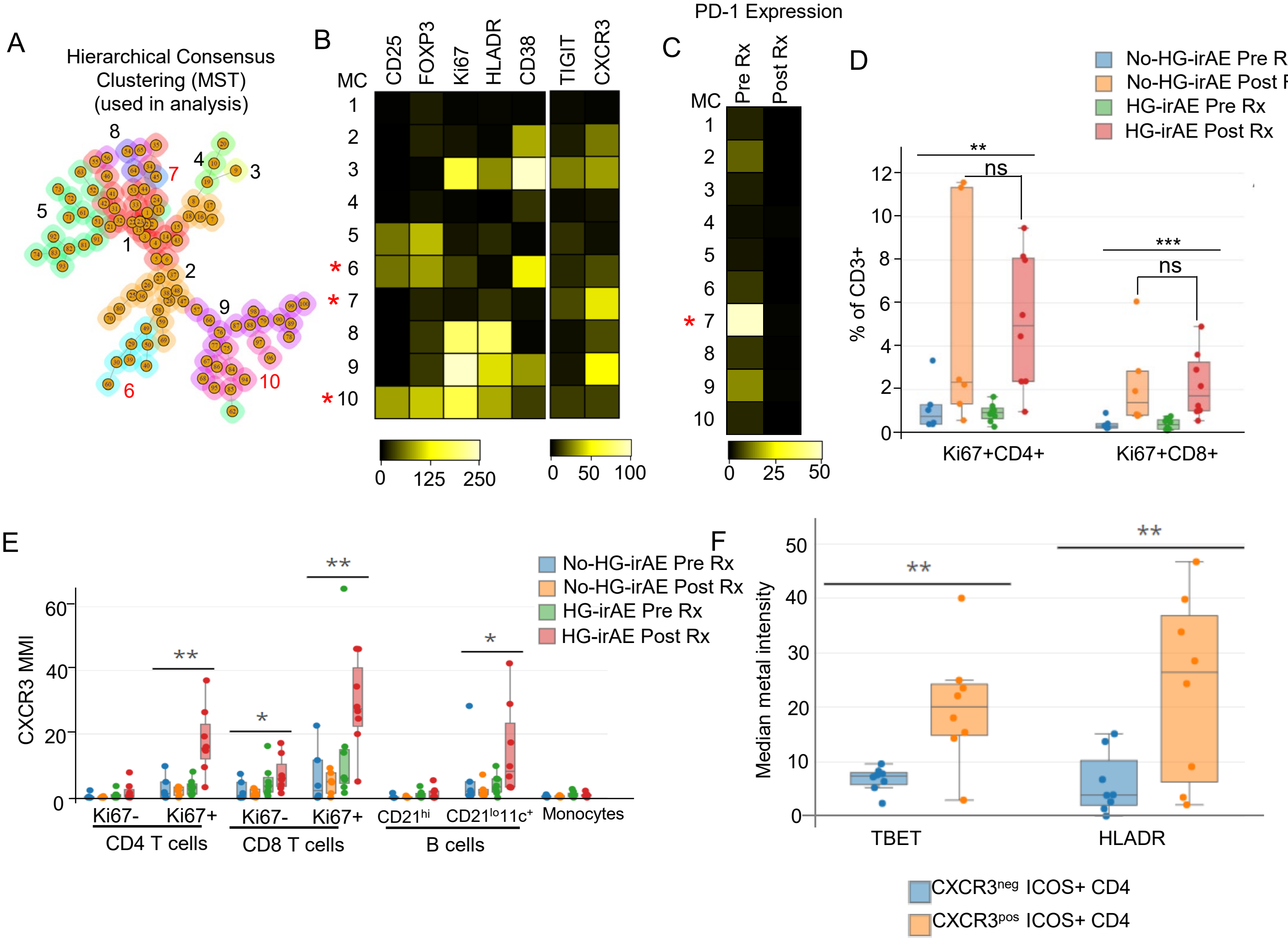

Suppl Fig3. Phenotypic/functional profiles of T cells. **(A-C)** FlowSOM clustering analysis performed on ICOS+CD4+ T cells revealed 10 distinct metaclusters (MC). **(A)** Hierarchical clustering used in the analysis. **(B)** Heatmap showing phenotype of the metaclusters. **(C)** Detection of surface PD-1 on the ICOS+CD4+ T cell metaclusters pre and post therapy with CCB. **(D)** Proportion of proliferating (Ki-67+) CD4+ and CD8+ T cells in pre and post treatment patient samples. **$p<0.01$, ***$p<0.001$, by Kruskal-Wallis test and ns=not significant by Mann-Whitney. **(E)** Correlation between changes in CXCR3 expression (median metal intensity or MMI) on various cell populations before/after therapy in patients with/without HG-irAEs. *$p<0.05$, **$p<0.01$, Kruskal Wallis. **(F)** Expression of T-bet and HLA-DR on CXCR3+ICOS+CD4 T cells expanded in patients with HG-irAE, as compared to the CXCR3-ICOS+CD4 T cell counterparts in these patients. **$p<0.01$, Wilcoxon matched-pairs signed rank test.

Supplementary online Methods

Patients and specimens:
Blood specimens were obtained from melanoma patients receiving combination checkpoint blockade following informed consent approved by institutional review board. Peripheral blood mononuclear cells (PBMCs) were obtained utilizing Ficoll density gradient centrifugation, as described previously(1).

Sex as a biological variable:
Study included both male and female patients with melanoma.

Single cell mass cytometry
PBMCs were thawed and stained with metal conjugated antibodies following manufacturer's recommendations as previously described(1). Briefly, cells were labeled with extracellular antibodies to detect cell surface proteins followed by fixation and permeabilization for intracellular/intranuclear labeling using intracellular antibodies. Cells were stained with cisplatin to detect viability and were incubated with Intercalator-Ir prior to analysis. Data were analyzed using Cytobank analysis software (Beckman Coulter Life Sciences).

Human T:B cocultures
B cells were isolated from PBMCs via immunomagnetic sorting using CD19+ beads (Miltenyi). Flow-sorted ICOS+/- CD4 T cells were cultured with B cells at a ratio of 1:10 (20,000 T cells:200,000 B cells) in 100 uL medium (cRPMI + 10% FBS) with SEB (1 ug/mL, VWR) and LPS (5 ug/mL, MD Bioproducts) for 1 week, based on methods adapted from prior studies(2, 3). Supernatant was collected for immunoglobulin analysis via ELISA (Bethyl Laboratories). After one week, cells were harvested and B cell phenotypes were analyzed via flow cytometry. In some experiments, either memory or naïve B cells were isolated using immunomagnetic sorting kits (Miltenyi) and cultured with patient ICOS+/- CD4 T cells. For some experiments Tregs (CD4+CD127^{negative} CD25^{hi} cells) were depleted from ICOS+CD4+ T cells prior to T:B coculture. For blocking experiments, 10ug/mL of anti-CD40 (clone 82102, R&D systems), 20 ug/mL of anti-IFNg (clone 25718, R&D Systems), 20 ug/mL of sIL-21R (R&D Systems), or isotype controls (IgG2B, IgG2A, R&D Biosystems or IgG1, Biolegend) were added at the start of the coculture.

Flow cytometry and cell sorting
Cryopreserved PBMCs were thawed and rested for 1 hour in cRPMI + 5% pooled human serum prior to sorting. To sort CD4+ T cell populations for T:B cocultures and in vivo experiments, cells were stained with Live/Dead® Fixable Dead stain from Thermo Fisher Scientific followed by antibodies to detect: ICOS (clone C398.4A), CD127 (clone A019D5), CD25 (clone M-A251), CD4 (clone RPA-T4), CD3 (clone SK7), CD19 (clone HIB19)(Biolegend), and CD8 (clone SK1)(BD Biosciences). Cells were sorted using BD FACSAria..

To characterize B cell phenotypes after coculture, cells were harvested, washed with PBS and stained for 30 minutes at 4°C using the following markers: CD3 (clone HIT3a), CD38 (clone HIT2), CD19 (clone HIB19)(Biolegend), IgD (clone IA6-2), and CD27 (clone M-T271)(BD Biosciences). For in vivo studies, mouse cells were gated out using Ter119 (clone TER119)(BD Biosciences), mCD45 (clone 30-F11), and hCD45 (clone 2D1)(Biolegend). Live cells were identified using Live/Dead® Fixable Dead stain from Thermo Fisher Scientific. Samples were washed with cold PBS and acquired on a BDCelesta. Data was analyzed using FlowJo.

ELISA
Levels of IgG were measured in T:B coculture supernatant and in mouse serum using the Human IgG ELISA kits from Bethyl Laboratories following manufacturers protocol.

In vivo human T:B interactions in humanized mice
ICOS+/- CD4 T cells and B cells were isolated as previously described. Cells were resuspended in a 1:1 ratio in sterile PBS and 0.1-1 million cells were injected retro-orbitally into MISTRG6 humanized mice(4). After 2 weeks, mice were euthanized and phenotype of human B cells /plasmablasts in the spleen was analyzed by flow cytometry as previously described(4). Serum human immunoglobulins were analyzed using an ELISA kit, as previously described.

MesoScale Discovery Assay
U-PLEX custom immuno-oncology panel kits were used to measure supernatant cytokines (IFNγ, sCD40L, and IL-21) following manufacturer's recommendations. Sort-purified ICOS + CD4 T cells were cultured in the presence of 200 ng/mL PMA and 1 ug/mL Ionomycin for 24 hours. Following stimulation, supernatant was collected and used to measure cytokine secretion.

Statistics:
Specific tests utilized for all comparisons are noted in the legends for each figure panel.

Data Availability:
Supporting data for figures is included in supplementary materials appendix.

Acknowledgments:

This work was supported in part by funds from NIH CA238471, AR077926 to KMD. MVD is supported in part by funds from NIH (CA197603). KMD and MVD are also supported in part by funds from Specialized Center for Research (SCOR) award from the Leukemia and Lymphoma Society. Authors also acknowledge support of the Immune Monitoring Resource of Winship Cancer Institute (P30CA138292) and Dr R. Halaban (Yale) for help with collection of some samples (P50CA121974).

References:

1. Das R, Bar N, Ferreira M, Newman AM, Zhang L, Bailur JK, et al. Early B cell changes predict autoimmunity following combination immune checkpoint blockade. *J Clin Invest.* 2018;128(2):715-20.
2. Rao DA, Gurish MF, Marshall JL, Slowikowski K, Fonseka CY, Liu Y, et al. Pathologically expanded peripheral T helper cell subset drives B cells in rheumatoid arthritis. *Nature.* 2017;542(7639):110-4.
3. Caielli S, Veiga DT, Balasubramanian P, Athale S, Domic B, Murat E, et al. A CD4(+) T cell population expanded in lupus blood provides B cell help through interleukin-10 and succinate. *Nat Med.* 2019;25(1):75-81.
4. Das R, Strowig T, Verma R, Koduru S, Hafemann A, Hopf S, et al. Microenvironment-dependent growth of preneoplastic and malignant plasma cells in humanized mice. *Nat Med.* 2016;22(11):1351-7.
